# Older people living with HIV: a population with high prevalence and severity of metabolic dysfunction-associated steatotic liver disease ‒ A cross-sectional study

**DOI:** 10.1016/j.bjid.2026.105967

**Published:** 2026-09-15

**Authors:** Rafaella Italiano Peixoto, Andrea Dória Batista, Armando Ykaro Soares de Oliveira, Ana Lúcia Coutinho Domingues, Paulo Sérgio Ramos de Araújo

**Affiliations:** a Universidade Federal de Pernambuco (UFPE), Hospital das Clínicas, Recife, PE, Brazil; b Universidade Federal de Pernambuco (UFPE), Centro de Ciências Médicas (CCM), Postgraduate Program in Tropical Medicine, Recife, PE, Brazil; c Universidade Federal de Pernambuco (UFPE), Faculdade de Medicina, Centro de Ciências Médicas (CCM), Department of Medicine, Recife, PE, Brazil; d Fundação Oswaldo Cruz (FIOCRUZ), Instituto Aggeu Magalhães, Recife, PE, Brazil

**Keywords:** HIV, Fatty liver, Non-alcoholic fatty liver disease, MASLD, NAFLD

## Abstract

**Background:**

Among People Living With Human immunodeficiency virus (PLWH) chronic liver disease is a major cause of non-HIV-related death. Factors related to HIV and its treatment may amplify the impact of metabolic risk factors and aging, thus leading to a high frequency of Metabolic Dysfunction-Associated Steatotic Liver Disease (MASLD) among this population. This study aimed to evaluate the frequency and associated factors of MASLD and its severity in older PLWH, and secondarily, to assess the frequency of MASLD-related fibrosis.

**Methods:**

This cross-sectional study included older PLWH followed at a referral center in northeastern Brazil. Hepatic steatosis was assessed by abdominal ultrasonography and liver fibrosis by point Shear-Wave Elastography (pSWE). Multivariable Poisson regression was performed to identify factors independently associated with hepatic steatosis and steatosis severity.

**Results:**

Ninety-two older PLWH were included. Hepatic steatosis was identified in 53.3% (n = 49) of participants, of whom 29 (53%) had moderate-to-severe steatosis. Significant fibrosis (F2 or greater) was identified in 10.6% of participants with steatosis who underwent elastography. Increased waist circumference, higher body mass index, metabolic syndrome, higher HbA1c and triglyceride levels were associated with hepatic steatosis. In multivariate analysis, increased waist circumference remained the factor most strongly associated with hepatic steatosis (PR = 4.69; 95% CI 1.53–14.38; p = 0.007). Factors associated with moderate-to-severe steatosis included metabolic and social factors, higher Alanine Aminotransferase (ALT) levels, dolutegravir use, absence of darunavir exposure and higher CD4 nadir. After adjustment, lipodystrophy (aPR = 1.63; 95% CI 1.04–2.56; p = 0.033) and higher Alanine Aminotransferase (ALT) levels (aPR = 1.03; 95% CI 1.01–1.04; p = 0.001) remained independently associated with greater steatosis severity.

**Conclusions:**

MASLD was highly prevalent among older PLWH, with a substantial proportion presenting moderate-to-severe steatosis and significant fibrosis. Metabolic and adiposity-related factors were independently associated with hepatic steatosis, while lipodystrophy and higher ALT levels were associated with greater steatosis severity.

## Graphical abstract

**Figure:**
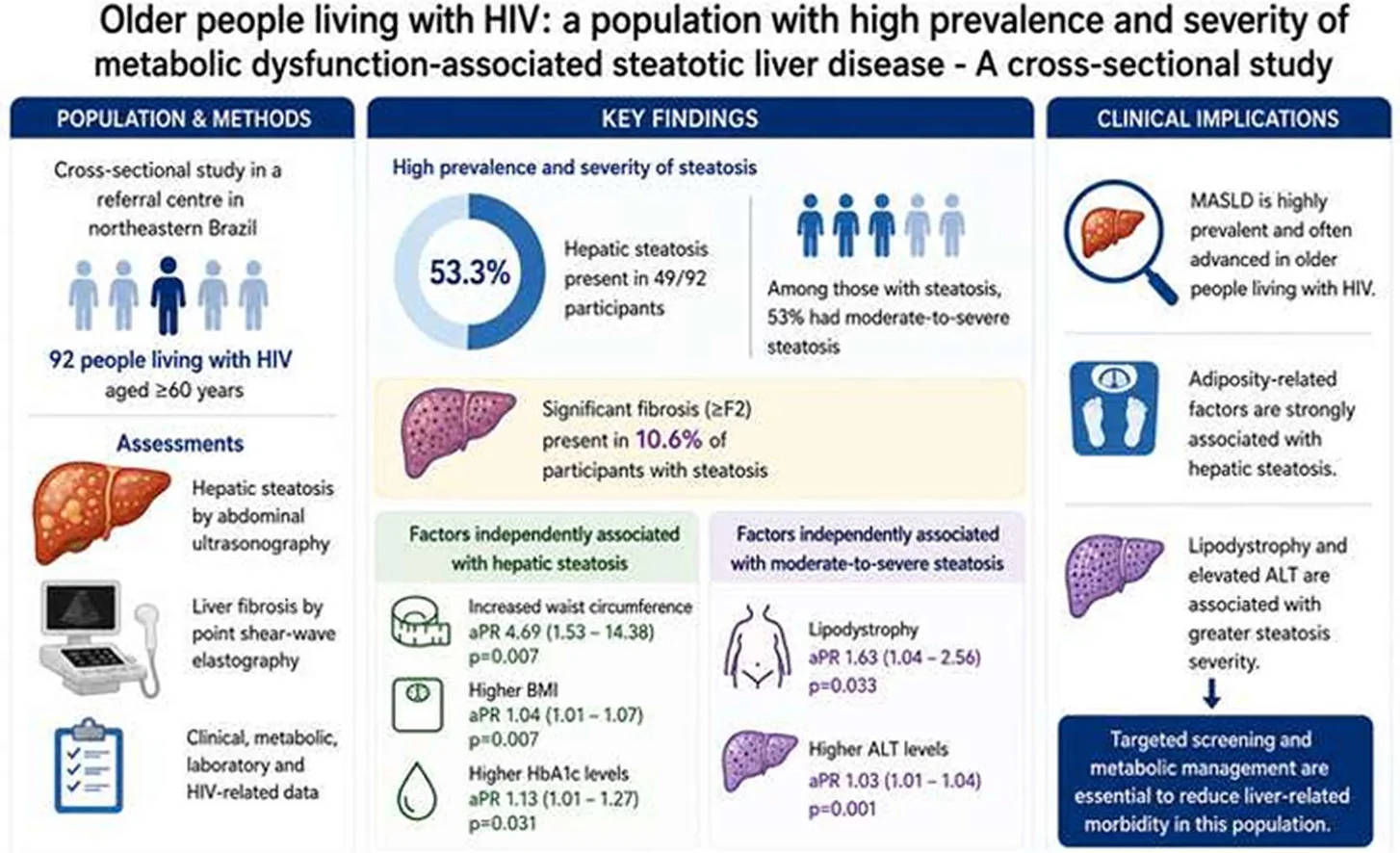

## Introduction

Increased rates of survival among People With Human Immunodeficiency Virus (PLWH) have led to the progressive aging of this population. Researchers have estimated that by 2030, 73% of patients with Human Immunodeficiency Virus (HIV) in Europe will be 50-years of age or older and that 84% of these individuals will have at least one comorbidity that is unrelated to Acquired Immunodeficiency Syndrome (AIDS).^1^ Among PLWH aged 60-years or older in Europe and North America, Chronic Liver Disease (CLD) is the fourth leading cause of non-HIV-related death, after neoplasms, cardiovascular diseases, and non-HIV-related infections.^2^ One of the main etiologies of CLD among PLWH is Metabolic Dysfunction-Associated Steatotic Liver Disease (MASLD).^3^

The global prevalence of MASLD is currently approximately 38% and continues to rise, along with its associated burden.^4^ Forecast models estimate that between 2020 and 2050, the number of cases of decompensated cirrhosis will more than triple, whereas the number of incident cases of liver cancer will nearly double, and the need for liver transplants will increase by a factor of nearly four.^5^

The prevalence of MASLD among PLWH has reached nearly 40%.^3^ The main risk factors for Steatotic Liver Disease (SLD) in this population are related to metabolic syndrome, such as obesity, Diabetes Mellitus (DM) and dyslipidemia.^6^ However, certain factors associated with hepatic steatosis are unique to PLWH, such as the presence of lipodystrophy, exposure to certain Antiretroviral Drugs (ARVs) and immunological changes resulting from HIV itself.^7^ In a cohort study from the United States, 43% of PLWH with MASLD had liver fibrosis of any grade at baseline biopsy, and 38% experienced fibrosis progression after one year. The mean fibrosis progression rate was approximately 0.2 stages per year, substantially higher than that reported in population-based studies of individuals without HIV, in whom fibrosis typically progresses at approximately 0.03 stages per year.^8^ These findings suggest that PLWH may be at increased risk of accelerated liver fibrosis progression compared with the general population.

Drugs such as stavudine (D4T) and Didanosine (DDI), which are associated with mitochondrial toxicity, have been associated with an increased prevalence of steatosis.^7^ Integrase Strand Transfer Inhibitors (INSTIs), such as Dolutegravir (DTG), have been associated with weight gain and increased visceral fat, which may contribute to liver disorders.^7^^,^^9^ However, controversies regarding the isolated role played by each antiretroviral class remain ongoing, and studies have reported different findings depending on the type of exposure, duration of use and association with other clinical and metabolic variables.

Recent literature highlights that, despite advances in the recognition of MASLD among PLWH, important knowledge gaps remain regarding its epidemiology, natural history, diagnosis, and treatment.^7^ Despite the increasing scientific production on hepatic steatosis among PLWH, studies that focus specifically on the older population remain lacking, especially in low and middle-income countries. Understanding the magnitude of the problem among older PLWH and identifying the associated factors can provide essential guidance for screening, prevention and management strategies.

The main objective of this study was to evaluate the frequency and severity of hepatic steatosis and the factors associated with its presence among PLWH aged 60-years or older. The secondary objective was to assess the frequency of MASLD-associated fibrosis.

## Materials and methods

### Study design

This analytical cross-sectional study included PLWH receiving follow-up care at the infectious diseases outpatient clinic of *Hospital das Clínicas, Federal University of Pernambuco* (HC/UFPE), a regional referral center in northeastern Brazil.

Potential participants were identified through an active search of the outpatient clinic database. Individuals aged 60-years or older who met the predefined eligibility criteria were contacted by telephone and invited to participate in the study. Those who agreed to participate attended a dedicated study visit, during which eligibility was confirmed and clinical, laboratory, and imaging assessments were performed. Individuals with active Hepatitis B (HBV) or Hepatitis C (HCV) coinfection, notable frailty (defined as inability to walk) or alcohol consumption ≥ 50 g/day (350 g/week) for women and ≥ 60 g/day (420 g/week) for men were excluded to avoid enrolling individuals with Alcohol-related Liver Disease (ALD).^10^ Individuals with decompensated Heart Failure (HF), previously treated viral hepatitis (HBV or HCV), liver malignancy (primary or metastatic), or schistosomiasis with moderate or severe periportal fibrosis were excluded from the analysis of fibrosis assessed by point Shear-Wave Elastography (pSWE).

### Data collection

Data collection was performed based on structured interviews and through consultation of medical records. Furthermore, standardized instruments were used to record sociodemographic information, lifestyle habits, geriatric evaluation, anthropometric data, clinical history, current and past use of antiretroviral drugs and laboratory tests. Lipodystrophy was defined based on self-reported previous diagnosis, self-reported body fat redistribution suggestive of lipodystrophy (including fat accumulation in the posterior cervical region and abdomen and fat loss in the face, gluteal region, or limbs), or documentation of lipodystrophy in the medical records. Alcohol consumption was assessed through a structured face-to-face interview. Participants were asked about the type of alcoholic beverage, drinking frequency, and the average amount consumed per week. Weekly alcohol intake was converted into grams of ethanol using the standard drink definition of 14 g of ethanol (one serving of spirits, one can of beer, or one glass of wine).

The data referenced in this study were collected and managed with the assistance of Research Electronic Data Capture (REDCap) electronic data capture tools hosted by UFPE. Clinical and laboratory data were obtained from medical records and the Brazilian national HIV information systems for laboratory monitoring (SISCEL) and antiretroviral drug dispensation (SICLOM).

The cardiometabolic criteria considered in this study were based on the 2009 harmonized International Diabetes Federation (IDF) definition of metabolic syndrome, which defines metabolic syndrome as the presence of at least three of the following components: increased waist circumference (> 90 cm in men and > 80 cm in women, according to cutoffs for Latin American populations), elevated fasting glucose (≥ 100 mg/dL), previous diagnosis of type 2 diabetes or glucose-lowering treatment, elevated blood pressure (systolic ≥ 130 mmHg and/or diastolic ≥ 85 mmHg, or antihypertensive treatment), elevated triglycerides (≥ 150 mg/dL or lipid-lowering treatment), and reduced HDL cholesterol (≤ 40 mg/dL in men and ≤ 50 mg/dL in women).^11^

### Ultrasound evaluation

Ultrasound evaluation was performed by two medical researchers who have extensive experience and training in abdominal imaging. The classification of hepatic steatosis was performed based on traditional ultrasound criteria, considering the increase in hepatic echogenicity in comparison with the right renal cortex, the posterior attenuation of the ultrasound beam and the difficulty of visualizing the intrahepatic vessels and diaphragm. Steatosis was classified as mild, moderate or severe in accordance with these parameters.

To evaluate liver fibrosis, elastography was performed using the pSWE technique, with the goal of evaluating the Liver Stiffness Measure (LSM). In this context, a 3.5 MHz convex transducer was used, alongside SIEMENS ACUSON-2000 equipment. A total of 10 valid measurements were obtained in the right hepatic lobe, and the median Shear Wave propagation Velocity (SWVmed) was recorded. Exams with a variability index (IQR/median) of <15% and a success rate greater than 80% were considered as criteria for adequate technical quality of the exam.^12^ The LSM values were interpreted according to the criteria associated with the METAVIR system, in which context the cut-off points for shear wave velocity were as follows: F0‒F1 (velocity < 1.34 m/s), F2 (≥ 1.34 m/s), F3 (≥ 1.55 m/s) and F4 (≥ 1.80 m/s). Significant fibrosis was defined as a fibrosis stage of F2 or higher.^13^

### Statistical analysis

Statistical analysis was conducted with the assistance of STATA/SE version 12.0 and Microsoft Excel 365 software. All tests were performed at a significance level of 5% (p ≤ 0.05), and only valid answers were considered, whereas missing or ignored data were excluded. The results are presented in the form of tables, which include absolute and relative frequencies for categorical variables, alongside measures of central tendency and dispersion for numerical variables.

To verify the associations among the categorical variables, Pearson's Chi-Square test or, where appropriate, Fisher's exact test was applied. The normality of the quantitative variables was assessed by performing the Shapiro-Wilk test. For comparisons between two independent groups, Student's *t*-test was used when the data were normally distributed, and the Mann-Whitney test was used when the data were nonnormally distributed. In comparisons involving more than two groups, an Analysis of Variance (ANOVA) was conducted for normally distributed data, and the Kruskal-Wallis test was used in other cases.

In the bivariate analysis, variables with a p-value ≤ 0.20 were considered for inclusion in the multivariable model. Multivariable analysis was performed using Poisson regression with robust variance, and all selected variables were entered simultaneously into the model. The final selection of variables was based on the strength of the bivariate association, biological plausibility, previous literature, and the authors' clinical judgment, while considering the sample size to reduce the risk of model overfitting. Missing data were assessed for all variables included in the analyses. As the proportion of missing data was low across all variables, complete-case analysis was performed, and no imputation procedures were applied.

### Ethical aspects

The present study was approved by the Research Ethics Committee of HC/UFPE under protocol numbers 5,958169 and CAAE 74,119,223.1.0000.8807. All participants signed a free and Informed Consent Form (ICF) before their inclusion in the study.

## Results

### Descriptive analysis

**Fig. 1:**
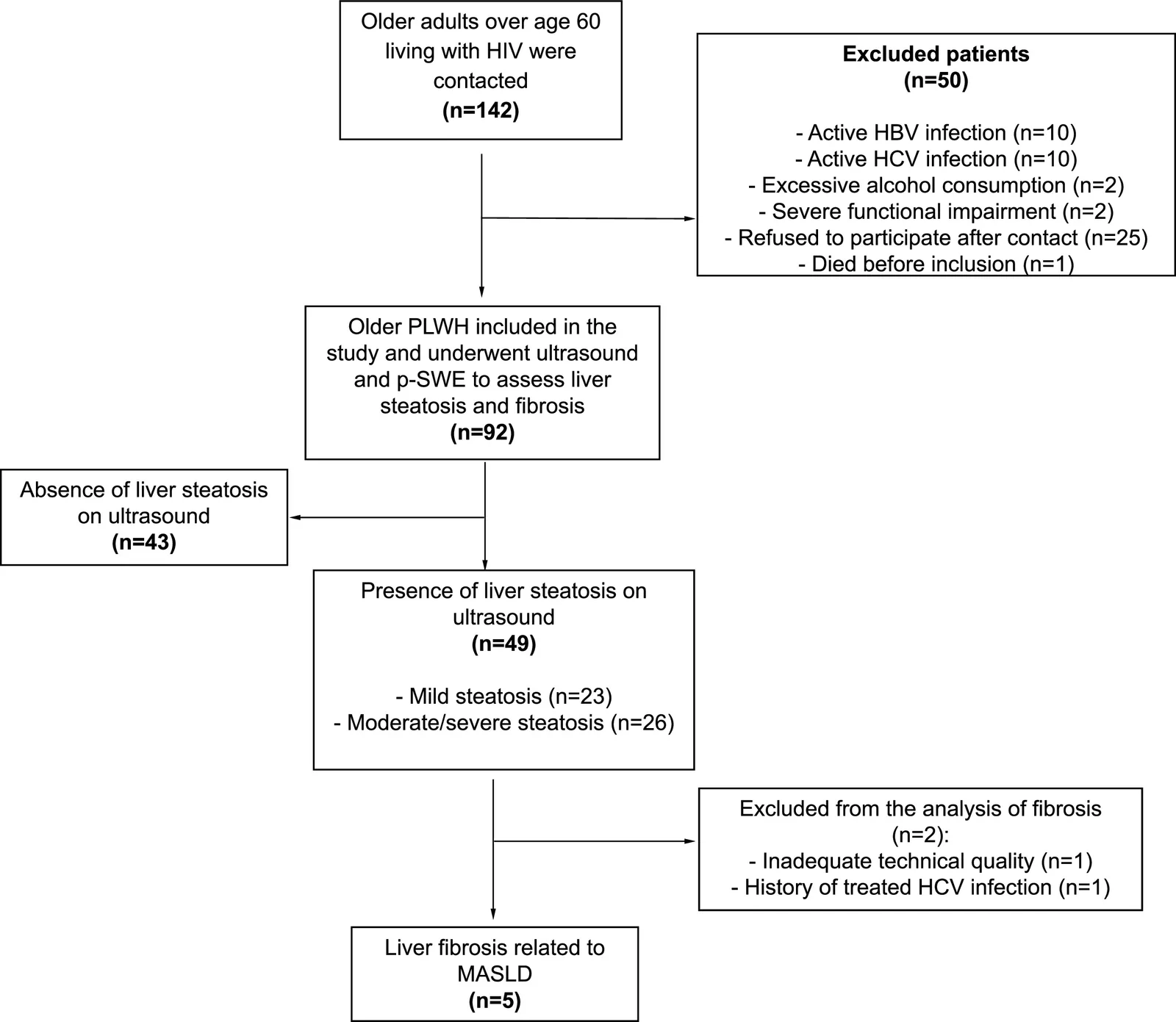
**Flow diagram of the study population.** PLWH, People Living With HIV; HBV, Hepatitis B Virus; HCV, Hepatitis C Virus; p-SWE, point Shear-Wave Elastography; MASLD, Metabolic Dysfunction-Associated Steatotic Liver Disease.

The final study sample consisted of 92 older PLWH, who were evaluated from January to July 2024. All patients in the final sample underwent ultrasound and p-SWE to assess hepatic steatosis and fibrosis (Fig. 1).

Table 1 presents the demographic, clinical, laboratory, and ultrasonographic characteristics of the 92 older PLWH according to the presence and severity of hepatic steatosis. Table 2 presents the HIV-related characteristics of the 92 older PLWH according to the presence and severity of hepatic steatosis.

**Table 1:** Demographic, clinical, and ultrasonographic characteristics of older PLWH by presence and grade of hepatic steatosis.

Characteristic	Overall (n = 92)	Hepatic steatosis		p-value	Grade of hepatic steatosis		p-value
		Yes (n = 49)	No (n = 43)		Mild (n = 23)	Moderate/ Severe (n = 26)	
Age, y	64.15 [62.6‒67.35]	63.6 [62.6‒67.85]	64.4 [62.4‒67.2]	0.978	64.6 [62.8‒71]	63.5 [62.32‒65.97]	0.111
Sex (male)	55 (59.8)	27 (55.1)	28 (65.1)	0.328	12 (52.2)	15 (57.7)	0.698
Race/ethnicity				0.807			0.667
White	36 (39.1)	18 (36.7)	18 (41.9)		8 (34.8)	10 (38.5)	
Black	12 (13)	6 (12.2)	6 (14)		4 (17.4)	2 (7.7)	
Brown (Pardo)	44 (47.8)	25 (51)	19 (44.2)		11 (47.8)	14 (53.8)	
Physical activity				0.748			0.770
Does not exercise	59 (64.1)	31 (63.3)	28 (65.1)		15 (65.2)	16 (61.5)	
1–2 times per week	11 (12)	7 (14.3)	4 (9.3)		4 (17.4)	3 (11.5)	
≥3 times per week	22 (23.9)	11 (22.4)	11 (25.6)		4 (17.4)	7 (26.9)	
Independent in ADLs	90 (97.8)	48 (98)	42 (97.7)	1.000	22 (95.7)	26 (100)	0.469
FRAIL Scale				0.222			0.892
Robust	67 (72.8)	35 (71.4)	32 (74.4)		16 (69.6)	19 (73.1)	
Pre-frail	16 (17.4)	11 (22.4)	5 (11.6)		5 (21.7)	6 (23.1)	
Frail	9 (9.8)	3 (6.1)	6 (14)		2 (8.7)	1 (3.8)	
Weight, kg	69 [60.5‒76.4]	73.2 [64.95‒85.1]	65.4 [53.6‒70]	**<0.001**	66 [63‒74.7]	76.4 [69.6‒89.9]	**0.003**
BMI, kg/m^2^	25.2 [22.5‒28.3]	27.2 [24.4‒32.52]	23 [20.5‒25.5]	**<0.001**	26 [24.4‒28.3]	28.4 [25.05‒33.75]	0.083
Waist circumference, cm	93.17 ± 13.26	99.8 ± 11.28	85.63 ± 11.22	**<0.001**	95.59 ± 8.84	103.52 ± 12.04	**0.012**
Calf, cm	34.25 [32‒36.5]	35.45 ± 2.83	33.39 ± 3.37	**0.002**	34.8 ± 2.65	36.02 ± 2.93	0.136
Metabolic syndrome^a^	70 (76.1)	42 (85.7)	28 (65.1)	**0.021**	16 (69.6)	26 (100)	**0.003**
Increased waist	62 (67.4)	45 (91.8)	17 (39.5)	**<0.001**	20 (87)	25 (96.2)	0.330
Hypertension	62 (67.4)	36 (73.5)	26 (60.5)	0.184	14 (60.9)	22 (84.6)	0.060
Dysglycemia	53 (57.6)	31 (64.6)	22 (51.2)	0.195	11 (50)	20 (76.9)	0.052
Hypertriglyceridemia	71 (77.2)	41 (83.7)	30 (69.8)	0.113	15 (65.2)	26 (100)	**0.001**
Low HDL	41 (44.6)	25 (51)	16 (37.2)	0.184	9 (39.1)	16 (61.5)	0.117
CV disease	13 (14.1)	7 (14.3)	6 (14)	0.964	4 (17.4)	3 (11.5)	0.692
Glucose, mg/dL	102 [94‒121.5]	107 [97‒132]	96 [93‒115]	**0.003**	105 [94‒127]	108.5 [101‒134.5]	0.235
HbA1c, %	5.5 [4.9‒6]	5.8 [4.9‒6.8]	5.2 [4.85‒5.7]	**0.008**	5.7 [4.7‒7]	5.9 [4.95‒6.7]	0.863
ALT, U/L	23.5 [20‒29.75]	25 [20.5‒33]	22 [19‒27]	0.059	22 [19‒26]	28.5 [23.5‒38]	**0.004**
AST, U/L	29 [25‒35]	29 [24‒35]	30 [26‒34]	0.832	29 [24‒34]	29.5 [26.5‒38.75]	0.125
Platelets, 10⁹/L	216.91 ± 51.95	228 ± 50.04	204.28 ± 51.76	**0.028**	232.83 ± 50.67	223.73 ± 50.07	0.531
Total cholesterol, mg/dL	201.15 ± 47.03	206.83 ± 49.46	194.67 ± 43.77	0.218	218.08 ± 55.28	196.88 ± 42.3	0.136
HDL cholesterol, mg/dL	46 [37.25‒58.75]	42 [36‒58.5]	47 [39‒60]	0.404	50 [36‒58]	39.5 [35.25‒59.25]	0.336
Triglycerides, mg/dL	150 [110‒222]	182.5 [131.75‒263]	130 [88‒177]	**0.040**	178 [111.3‒255]	184 [138‒311.5]	0.349
FIB-4	1.76 [1.49‒2.27]	1.66 [1.4‒2.21]	1.92 [1.64‒2.32]	0.090	1.66 [1.33‒2.19]	1.6 [1.4‒2.24]	0.881
SWV med, m/s^b^	1.13 [0.97‒1.27]	1.07 [0.95‒1.19]	1.17 [1.03‒1.4]	**0.010**	1.03 [0.9‒1.19]	1.08 [0.95‒1.19]	0.882

Data are presented as n (%), means ± SD, or medians [P25‒P75] according to distribution. Statistical significance was set for a value of p < 0.05. In bold: p < 0.05.

a According to the IDF criteria for metabolic syndrome (ALBERTI et al., 2009).

b Median SWV using point Shear-Wave Elastography (pSWE) analysis of 82 patients considering the exclusion of 10 patients according to the methodology BMI, Body Mass Index; HDL, High-Density Lipoprotein Cholesterol; ADLs, Activities of Daily Living; ALT, Alanine Aminotransferase; AST, Aspartate Aminotransferase; CV, Cardiovascular; FIB-4, Fibrosis-4 index; SWV, Shear Wave Velocity.

**Table 2:** HIV-related characteristics of older PLWH by presence and grade of hepatic steatosis.

Characteristic	Overall (n = 92)	Hepatic steatosis		p-value	Grade of hepatic steatosis		p-value
		Yes (n = 49)	No (n = 43)		Mild (n = 23)	Moderate/ Severe (n = 26)	
HIV diagnosis, y	16.84 ± 8.87	17.22 ± 7.76	16.42 ± 10.07	0.674	16.23 ± 7.07	18.09 ± 8.36	0.408
Age at HIV diagnosis, y	48.87 ± 10.32	48.51 ± 9.18	49.28 ± 11.57	0.726	50.49 ± 8.86	46.76 ± 9.28	0.158
HIV diagnosis before age 60	77 (83.7)	44 (89.8)	33 (76.7)	0.091	21 (91.3)	23 (88.5)	1.000
Lipodystrophy, n (%)	14 (15.2)	8 (16.3)	6 (14)	0.752	1 (4.3)	7 (26.9)	0.052
Time since VL suppression, y	9.55 [6.1‒14.9]	9.45 [7‒14.4]	9.7 [4.4‒17.2]	0.837	9.4 [7.2‒13.6]	9.5 [6.4‒18.7]	0.680
Nadir CD4+, cells/μL	134 [39‒265]	143.5 [67‒247.7]	106 [32‒265.5]	0.346	84 [56.5‒175.5]	193 [83.75‒359.25]	**0.048**
Maximum VL, 10^3^ copies/mL	87.81 [7.23‒242.5]	96.4 [5.66‒225]	84 [7.59‒359.76]	0.512	169.7 [3.41‒387.5]	27 [5.9‒102.93]	0.050
Current ART							
NRTI^a^	77 (83.7)	42 (85.7)	35 (81.4)	0.576	19 (82.6)	23 (88.5)	0.692
NNRTI^b^	3 (3.3)	1 (2)	2 (4.7)	0.597	1 (4.3)	0 (0)	0.469
PI (DRV+RTV)	29 (31.5)	14 (28.6)	15 (34.9)	0.516	10 (43.5)	4 (15.4)	**0.030**
INSTI (DTG)	77 (83.7)	41 (83.7)	36 (83.7)	0.995	16 (69.6)	25 (96.2)	**0.019**
Past ART^c^							
NRTI	92 (100)	49 (100)	43 (100)	‒	23 (100)	26 (100)	-
AZT	66 (71.7)	36 (73.5)	30 (69.8)	0.694	17 (73.9)	19 (73.1)	0.947
ddI	18 (19.6)	7 (14.3)	11 (25.6)	0.173	3 (13)	4 (15.4)	1.000
d4T	12 (13)	5 (10.2)	7 (16.3)	0.388	2 (8.7)	3 (11.5)	1.000
TDF	71 (78.3)	35 (71.4)	37 (86)	0.090	16 (69.6)	19 (73.1)	0.786
NNRTI	62 (67.4)	35 (71.4)	27 (62.8)	0.378	19 (82.6)	16 (61.5)	0.103
EFV	52 (56.5)	32 (65.3)	20 (46.5)	0.070	17 (73.9)	15 (57.7)	0.234
NVP	13 (14.1)	4 (8.2)	9 (20.9)	0.079	2 (8.7)	2 (7.7)	1.000
PI	63 (68.5)	34 (69.4)	29 (67.4)	0.841	17 (73.9)	17 (65.4)	0.518
RTV	61 (66.3)	33 (67.3)	28 (65.1)	0.821	17 (73.9)	16 (61.5)	0.357
ATV	33 (35.9)	19 (38.8)	14 (32.6)	0.535	7 (30.4)	12 (46.2)	0.260
DRV	44 (47.8)	22 (44.9)	22 (51.2)	0.548	14 (60.9)	8 (30.8)	**0.035**
INSTI	80 (87)	43 (87.8)	37 (86)	0.808	17 (73.9)	26 (100)	**0.007**
DTG	80 (87)	43 (87.8)	37 (86)	0.808	17 (73.9)	26 (100)	**0.007**
RAL	9 (9.8)	3 (6.1)	6 (14)	0.296	2 (8.7)	1 (3.8)	0.594

Data are presented as n (%), means ±SD, or medians [P25‒P75] according to distribution. Statistical significance was set for a value of p < 0.05. In bold: p < 0.05.

a NRTI used in current ART: 3TC (n = 59) or 3TC+TDF (n = 15) or AZT+3TC (n = 1).

b NNRTI used in current ART: EFV (n = 1) or NVP (n = 2).

c Overall frequency of past use of other ARVs without statistical significance between groups = 3TC: 49 (100%); ABC: 11 (12%); ddC: 2 (2.2%); LPV: 21 (22.8%); NFV: 11 (12%); SQV: 3 (3.3%); IDV: 8 (8.7%); FPV: 2 (2.2%); T-20: 2 (2.2%); ETR: 1 (1.1%); MVC: 1 (1.1%). 3TC, Lamivudine; ABC, Abacavir; ART, Antiretroviral Therapy; ATV, Atazanavir; AZT, Zidovudine; d4T, Stavudine; ddC, Zalcitabine; ddI, Didanosine; DRV, Darunavir; DTG, Dolutegravir; EFV, Efavirenz; ETR, Etravirine; FPV, Fosamprenavir; IDV, Indinavir; INSTI, Integrase Strand Transfer Inhibitor; LPV, Lopinavir; MVC, Maraviroc; NFV, Nelfinavir; NNRTI, Non-Nucleoside Reverse Transcriptase Inhibitor; NRTI, Nucleoside Reverse Transcriptase Inhibitor; NVP, Nevirapine; PI, Protease Inhibitor; RAL, Raltegravir; RTV, Ritonavir; SQV, Saquinavir; T-20, Enfuvirtide; TDF, Tenofovir; VL, Viral Load.

According to the Lipschitz BMI classification for older adults, 20 participants (21.7%) were classified as underweight (BMI < 22 kg/m^2^), 38 (41.3%) had normal weight (BMI 22–27 kg/m^2^), and 34 (37.0%) were classified as overweight (BMI > 27 kg/m^2^). Regarding age distribution, 73 participants (79.3%) were aged 60–69 years, 18 (19.6%) were aged 70–79 years, and 1 (1.1%) was aged ≥ 80-years.

Most participants were functionally independent, with 97.8% being independent in Activities of Daily Living (ADLs). Regarding frailty status, 72.8% were classified as robust, 17.4% as pre-frail, and 9.8% as frail according to the FRAIL scale. Regarding antiretroviral treatment, 53.3% of the participants were receiving a regimen containing lamivudine (3TC) and DTG; 16.3% were receiving Darunavir with Ritonavir (DRV/r) plus DTG; 12% were receiving 3TC, Tenofovir (TDF) and DTG; 8.7% were receiving 3TC and DRV/r; 4.3% were receiving 3TC, TDF, and DRV/r. The remaining participants received various regimens that included Zidovudine (AZT), Nevirapine (NVP) or Efavirenz (EFV) in combination with 3TC, DTG, TDF or DRV/r.

### Descriptive analysis of the ultrasound results

Hepatic steatosis was identified in 53.3% of participants (n = 49). In the overall sample, steatosis was classified as mild in 23 participants (25.0%) and moderate-to-severe in 26 (28.3%) (moderate, n = 18; severe, n = 8).

Among older PLWH with hepatic steatosis, 46 (94%) reported low-risk alcohol consumption and had at least one cardiometabolic risk factor; therefore, these patients met criteria for MASLD.^10^ Three patients with SLD reported alcohol consumption between 20 and 50 *g*/day among women or between 30 and 60 *g*/day among men. These patients were classified as MASLD with increased alcohol intake (MetALD).^14^

Among participants with hepatic steatosis, two patients were excluded from the analysis of MASLD-associated fibrosis: one due to previously treated HCV infection and the other due to not meeting the technical quality criteria for hepatic elastography. Among the 47 individuals included in this analysis, 89.4% were classified as F0/F1, whereas 5 (10.6%) had significant fibrosis (≥ F2), including 4 with F2 fibrosis and 1 with F4 fibrosis. Regarding the FIB-4 score, among individuals with hepatic steatosis, 7 (14.3%) had FIB-4 values < 1.3, 38 (77.6%) had values between 1.3 and 2.67, and 4 (8.2%) had values > 2.67. None of the participants with values below 1.3 presented significant hepatic fibrosis on elastography.

### Analysis of the factors associated with hepatic steatosis

The presence of hepatic steatosis was significantly associated with several clinical and anthropometric factors: higher body weight, higher BMI values, increased waist circumference, increased calf circumference and the presence of metabolic syndrome. With respect to laboratory parameters, individuals with SLD exhibited higher fasting glucose levels, HbA1c levels, platelet counts and triglyceride levels.

Multivariable Poisson regression analysis was performed to evaluate factors independently associated with the presence of liver steatosis (Table 3). After adjustment, increased waist circumference, higher BMI, and higher HbA1c levels remained significantly associated with liver steatosis.

**Table 3:** Multivariate Poisson regression analysis of factors associated with hepatic steatosis in older PLWH.

Variables	n	Hepatic Steatosis	Unadjusted Analysis		Adjusted Analysis	
		n (%)	PR (95%CI)	p-value	aPR (95% CI)	p-value
**Increased waist circumference**						
Yes	62	45 (72.6)	5.44 (2.16 – 13.73)	<0.001^a^	4.69 (1.53 – 14.38)	0.007
No	30	4 (13.3)	1			
**Age of HIV diagnosis**				0.091^a^		
≥ 60	15	5 (33.3)	1			
< 60	77	44 (57.1)	1.71 (0.82 ‒ 8.54)			
**Past ART: NVP**						
Yes	13	4 (30.8)	1	0.079^a^		
No	79	45 (57)	1.85 (0.8 – 4.28)			
**Past ART: TDF**						
Yes	72	35 (48.6)	1	0.090^a^		
No	20	14 (70)	1.44 (0.99 – 2.09)			
**Past ART: EFZ**						
Yes	52	32 (61.5)	1.45 (0.95 – 2.2)	0.070^a^		
No	40	17 (42.5)	1			
**BMI**	91	‒	1.08 (1.05 ‒ 1.11)	<0.001^c^	1.04 (1.01 ‒ 1.07)	0.007
**HbA1c**	87	‒	1.21 (1.11 ‒ 1.32)	<0.001^c^	1.13 (1.01 ‒ 1.27)	0.031
**Triglycerides**	89	‒	1.00 (1.00 ‒ 1.00)	0.011^c^		

a Chi-square test; ** Fisher’s exact test;.

c Wald test. ART, Antiretroviral Therapy; BMI, Body Mass Index; EFV, Efavirenz; NVP, Nevirapine; TDF, Tenofovir; PR, Prevalence Ratio; aPR, Adjusted prevalence ratio; 95%CI, 95% Confidence Interval.

### Analysis of the factors associated with moderate-to-severe hepatic steatosis

Among 49 older PLWH with MASLD, 26 (53%) had moderate-to-severe steatosis. Moderate-to-severe hepatic steatosis was significantly associated with various clinical, demographic, anthropometric and laboratory factors (Table 1). Associations were observed between more intense steatosis and higher educational level, higher household income, increased body weight, increased waist circumference, metabolic syndrome, hypertriglyceridemia and higher ALT levels. Regarding HIV-related factors (Table 2), moderate-to-severe hepatic steatosis was associated with higher CD4 nadir and exposure to DTG, while an inverse association was observed with DRV exposure.

Multivariable Poisson regression analysis was performed to evaluate factors independently associated with moderate-to-severe hepatic steatosis (Table 4). After adjustment, lipodystrophy and higher ALT remained independently associated with moderate-to-severe hepatic steatosis.

**Table 4:** Multivariate Poisson regression analysis of factors associated with moderate-to-severe steatosis in older PLWH.

Variables	n	Moderate-to-severe steatosis	Unadjusted Analysis		Adjusted Analysis	
		n (%)	PR (95%CI)	p-value	aPR (95%CI)	p-value
Dysglycemia				0.052^a^		
Yes	31	20 (64.5)	1.83 (0.91 ‒ 3.69)			
No	17	6 (35.3)	1			
Lipodystrophy				0.052^b^		
Yes	8	7 (87.5)	1.89 (1.23 – 2.89)		1.63 (1.04 – 2.56)	**0.033**
No	41	19 (46.3)	1			
Current ART: Darunavir						
Yes	14	4 (28.6)	0.45 (0.19 – 1.09)	**0.030**^a^		
No	35	22 (62.9)	1			
Current ART: Dolutegravir						
Yes	41	25 (61)	4.88 (0.75 – 31.61)	0.019^b^		
No	8	1 (12.5)	1			
ALT	49	‒	1.03 (1.01 ‒ 1.04)	**<0.001**^c^	1.03 (1.01 – 1.04)	**0.001**

a Chi-square test;.

b Fisher’s exact test;.

c Wald test. ALT, Alanine Aminotransferase; ART, Antiretroviral Therapy; PR, Prevalence Ratio; aPR, Adjusted Prevalence Ratio; 95% CI, 95% Confidence Interval.

## Discussion

In the present study, hepatic steatosis was highly prevalent among older PLWH, affecting more than half of the study population (53.3%). This frequency is substantially higher than the prevalence of MASLD reported in the general Brazilian population (33.4%).^15^

Population-based studies have reported a high prevalence of MASLD among older adults. In the United States, the prevalence was 40.3% among individuals aged 60–74 years and 39.2% among those aged ≥ 75-years.^16^ MASLD prevalence has also been reported to decrease with advancing age, reaching 34.8% among individuals aged 70-years and older.^17^ As age increases, changes such as the redistribution of adipose tissue with central accumulation, the senescence of adipocytes and chronic inflammation also contribute to the increased prevalence of MASLD.^7^

The prevalence of MASLD among individuals with HIV monoinfection ranges from 33% to 43%, according to a recent meta-analysis.^3^ The higher frequency observed in the present study may be explained by the older age of the study population, which likely reflects prolonged cumulative exposure to both HIV infection and ART, alongside a higher prevalence of age-related comorbidities such as metabolic syndrome and diabetes mellitus. In our sample, 85% had metabolic syndrome and nearly two-thirds had diabetes mellitus.

In the present study, increased waist circumference emerged as the factor most strongly associated with hepatic steatosis, in agreement with previous studies.^18^^,^^19^

In our study, the frequency of lean MASLD, which is defined as the presence of hepatic steatosis in individuals with a BMI lower than 25 kg/m^2^, was 15.2%. These findings are comparable to those of a previous study, which reported a 13.9% prevalence of lean MASLD among PLWH.^20^ If we consider Lipschitz criterion for normal weight among older individuals (BMI <27 kg/m^2^) the prevalence of lean MASLD in the total sample size increases to 25%, accounting for nearly half of the SLD cases.^21^ These findings reinforce that a BMI within the normal range for older adults does not exclude the possibility of hepatic steatosis, given the high frequency of lean MASLD observed in this age group. Older age has also been identified as a factor associated with lean MASLD among PLWH.^22^

In terms of laboratory findings, individuals with MASLD exhibited higher fasting glucose, HbA1c, and triglyceride levels. Elevated triglyceride levels have previously been associated with MASLD among PLWH, consistent with the findings of our study.^3^^,^^19^

The American Diabetes Association (ADA) consensus highlights hyperglycemia as an important risk factor for MASLD. In our study, the prevalence of hepatic steatosis among individuals with DM was 64.6%.^23^

DTG use was associated with moderate-to-severe hepatic steatosis among the individuals included in our sample. These findings are consistent with previous studies linking DTG exposure to weight gain and an increased risk of MASLD among PLWH.^23^ Although boosted PIs have been associated with adverse metabolic effects, including dyslipidemia and increased visceral adiposity, PI use in our study was associated with mild steatosis.^7^ However, this finding did not persist after multivariable adjustment. Both findings should be interpreted with caution. Most participants (87%) were receiving DTG-based regimens, reflecting current Brazilian treatment recommendations, whereas only a small proportion remained on boosted PI or NNRTI-based regimens. This imbalance in antiretroviral exposure reduced the variability between treatment groups and may have limited comparisons across antiretroviral classes. This imbalance in antiretroviral exposure reduced the variability between treatment groups and could have limited comparisons across antiretroviral classes. The European AIDS Clinical Society (EACS) has highlighted the risk of weight gain induced by the INSTI class.^24^ Nevertheless, INSTI-based regimens remain the preferred first-line antiretroviral therapy because of their high efficacy and favorable resistance profile. Rather than discouraging their use, our findings reinforce the importance of regular metabolic screening and appropriate management of cardiometabolic risk factors in PLWH receiving INSTI-based therapy.

Among participants with hepatic steatosis, more than half presented moderate-to-severe steatosis, a proportion similar to that observed in a previous Australian case-control study (51.1%). In that study, more severe steatosis was associated with higher BMI, plasma HIV viral load, blood glucose levels, and a higher prevalence of lipodystrophy.^25^ In the present study, the association between moderate-to-severe steatosis and markers of adiposity and metabolic dysfunction reinforces the central role of metabolic derangement in the progression of liver steatosis among older PLWH. Elevated ALT levels also remained independently associated with more severe steatosis after multivariable adjustment, consistent with previous evidence linking ALT elevation to MASLD activity and steatohepatitis.^19^^,^^21^ These findings support the role of ALT as a clinically relevant marker for identifying individuals at higher risk of progressive liver disease.

Lipodystrophy was independently associated with moderate-to-severe steatosis in our study. This finding may reflect the long-term metabolic consequences of exposure to older antiretroviral drugs associated with mitochondrial toxicity, particularly Zidovudine (AZT). Mitochondrial dysfunction related to thymidine analogues may lead to persistent alterations in adipose tissue distribution and metabolic homeostasis, even years after treatment modification.^26^ Notably, all participants with lipodystrophy in our cohort had previous exposure to AZT.

Among individuals with hepatic steatosis who underwent liver elastography, 10.6% had significant fibrosis. This prevalence was similar to that observed in a previous study from the United States (13.9%) but lower than that reported in a study from Brazil (26.9%).^27^^,^^28^ Differences in fibrosis frequency across studies may be related to variations in age distribution, metabolic profile, duration of HIV infection and ART exposure, as well as differences in fibrosis assessment methods and diagnostic cut-off values.

The identification of MASLD-associated fibrosis is essential to guide therapeutic interventions and reduce the risk of adverse liver-related outcomes. The FIB-4 index is currently the most widely recommended noninvasive tool for initial fibrosis screening in this setting. Patients with values ≥ 1.3 should undergo additional evaluation with complementary methods such as Vibration-Controlled Transient Elastography (VCTE), 2D Shear-Wave Elastography (2D-SWE), pSWE, or magnetic resonance to improve risk stratification.^22^ In the present study, the high frequency of MASLD and significant liver fibrosis observed among older PLWH reinforces the need for systematic liver screening strategies in this population. Notably, none of the individuals with FIB-4 values below 1.3 had significant liver fibrosis on elastography.

### Study limitations

Since this study included only PLWH, survival bias may have influenced the findings. The sample likely consisted predominantly of long-term HIV survivors with a healthier clinical profile, which may have led to an underestimation of the true frequency of liver steatosis and fibrosis. Individuals with more severe HIV-related disease and metabolic complications may have died at earlier ages and therefore may have been underrepresented in the study population.

In addition, individuals who initiate ART early after diagnosis or who were less exposed to older antiretroviral drugs may experience a lower burden of age-related comorbidities than those exposed to prolonged untreated viremia and older ARV regimens. Among the 92 participants included in this study, only one had acquired HIV before the availability of combination ART, which may reflect the historically higher mortality observed in individuals diagnosed during that period.

Moreover, the absence of comparison groups restricts causal interpretation. Because only older people with HIV were included, it was not possible to disentangle the effects of aging, HIV infection, cumulative antiretroviral exposure, and conventional cardiometabolic risk factors on hepatic steatosis. Studies including younger people with HIV from the same geographic region would be useful to evaluate whether the prevalence and severity of steatosis increase with age under similar socioeconomic, environmental, and healthcare conditions. In parallel, studies with an HIV-negative control group matched for age, sex, and cardiometabolic profile could help estimate the independent contribution of HIV infection and antiretroviral exposure to hepatic steatosis and fibrosis. Multicenter studies with larger samples and appropriately selected comparison groups are therefore needed to confirm these findings and improve their generalizability.

Despite these limitations, an important strength of this study is the inclusion of older PLWH, a population historically underrepresented in studies evaluating MASLD. Notably, a high frequency of hepatic steatosis was observed even in this survivor population, suggesting that the burden of steatosis and fibrosis in the broader population of PLWH may be even greater than that observed in our sample.

## Conclusion

MASLD was highly prevalent among older PLWH, affecting 53.3% of the study population. Among individuals with hepatic steatosis, more than half presented moderate-to-severe disease, and 10.6% had clinically significant fibrosis on elastography. Metabolic and adiposity-related factors were independently associated with hepatic steatosis, while lipodystrophy and elevated ALT levels were associated with greater steatosis severity. These findings highlight the growing metabolic and hepatic burden among aging PLWH and reinforce the importance of targeted metabolic and liver assessment in this population.

## Abbreviations

3TC, Lamivudine; AIDS, Acquired Immunodeficiency Syndrome; ALD, Alcohol-related Liver Disease; ART, Antiretroviral Therapy; ARVs, Antiretrovirals; AZT, Zidovudine; ADL, Activities of Daily Living; BMI, Body Mass Index; CLD, Chronic Liver Disease; DM, Diabetes Mellitus; d4T, Stavudine; DDI (ddI), Didanosine; DRV, Darunavir; DRV/r, Darunavir boosted with Ritonavir; DTG, Dolutegravir; FIB-4, Fibrosis-4 Index; HBV, Hepatitis B Virus; HCV, Hepatitis C Virus; HIV, Human Immunodeficiency Virus; INSTI(s), Integrase Strand Transfer Inhibitor(s); LSM, Liver Stiffness Measure; MASLD, Metabolic dysfunction-Associated Steatotic Liver Disease; NVP, Nevirapine; PI, Protease Inhibitor; PLWH, People living with HIV; pSWE, Point Shear Wave Elastography; SLD, Steatotic Liver Disease; SWVmed: Median Shear Wave Velocity TDF, Tenofovir Disoproxil Fumarate; VL, Viral Load; VCTE, Vibration-Controlled Transient Elastography.

## Consent for publication

Not applicable.

## Data availability

The data that support the findings of this study are available from the corresponding author upon reasonable request.

## Ethics approval statement and patient consent statement

The present study was approved by the Research Ethics Committee of HC/UFPE under protocol numbers 5958,169 and CAAE 74,119,223.1.0000.8807. All participants signed a free and Informed Consent Form (ICF) before their inclusion in the study. This study was performed in accordance with the Declaration of Helsinki.

## Declaration of generative AI and AI-assisted technologies in the manuscript preparation process

During the preparation of this work, the authors used ChatGPT (OpenAI) in order to improve the clarity, grammar, and readability of the manuscript. After using this tool, the authors reviewed and edited the content as needed and take full responsibility for the content of the published article.

## Authors’ contributions

RIP, PSRA, and ADB conceived and designed the study. RIP, ADB, ALCD, and AYSO contributed to data acquisition. ADB and ALCD performed the ultrasonographic examinations. RIP analyzed the data and drafted the manuscript. PSRA, ADB, and ALCD critically revised the manuscript. All authors read and approved the final manuscript.

## Funding

This research did not receive any specific grant from funding agencies in the public, commercial, or not-for-profit sectors.

## Conflicts of interest

The authors declare no conflicts of interest.
